# Supplementary figures and images for: A mechanosensing mechanism controls plasma membrane shape homeostasis at the nanoscale
Source: eLife. 2023 Sep 25;12:e72316. doi: 10.7554/eLife.72316 (PMC10569792; doi:10.7554/eLife.72316)

10 second exposure

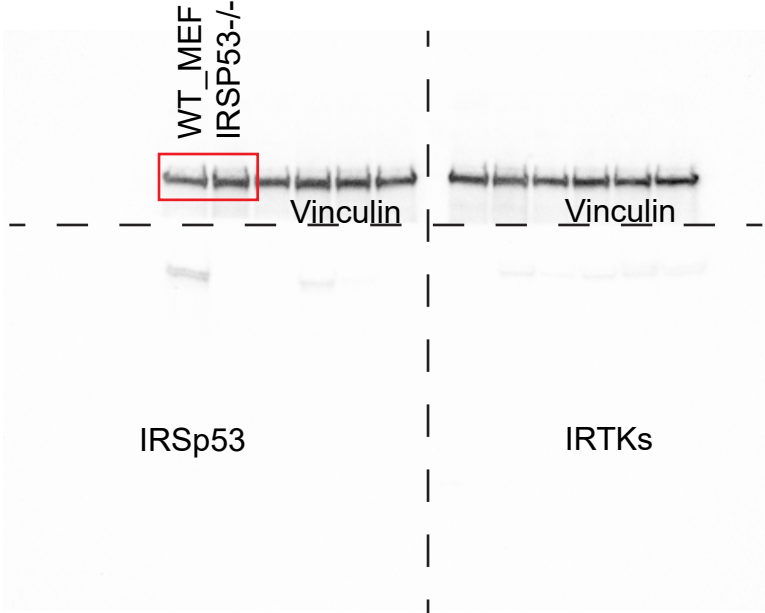

Marker overlay

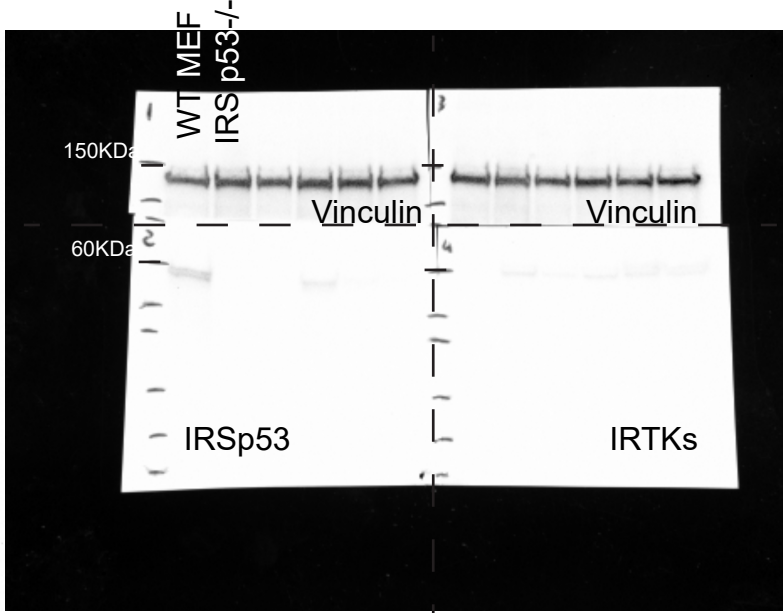

80 second exposure

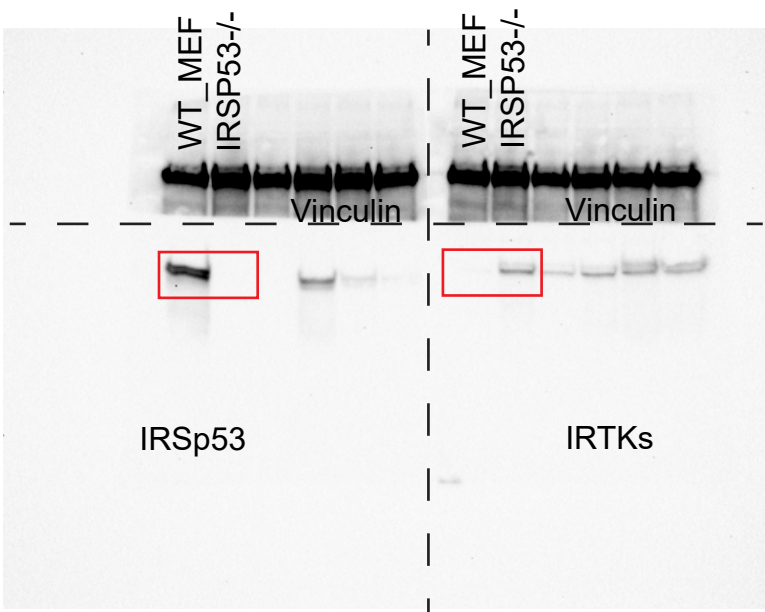

Marker overlay

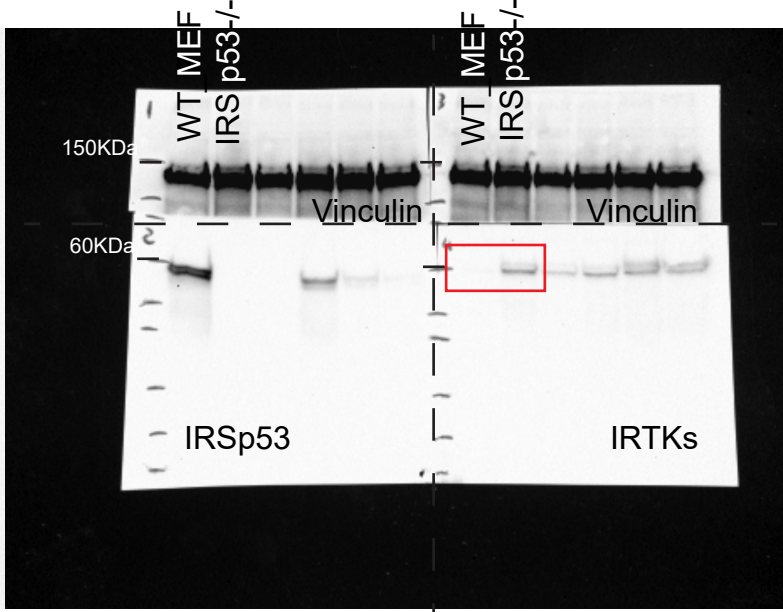

Supplement: Figure 2—figure supplement 1—source data 2. [file elife-72316-fig2-figsupp1-data2.pdf]

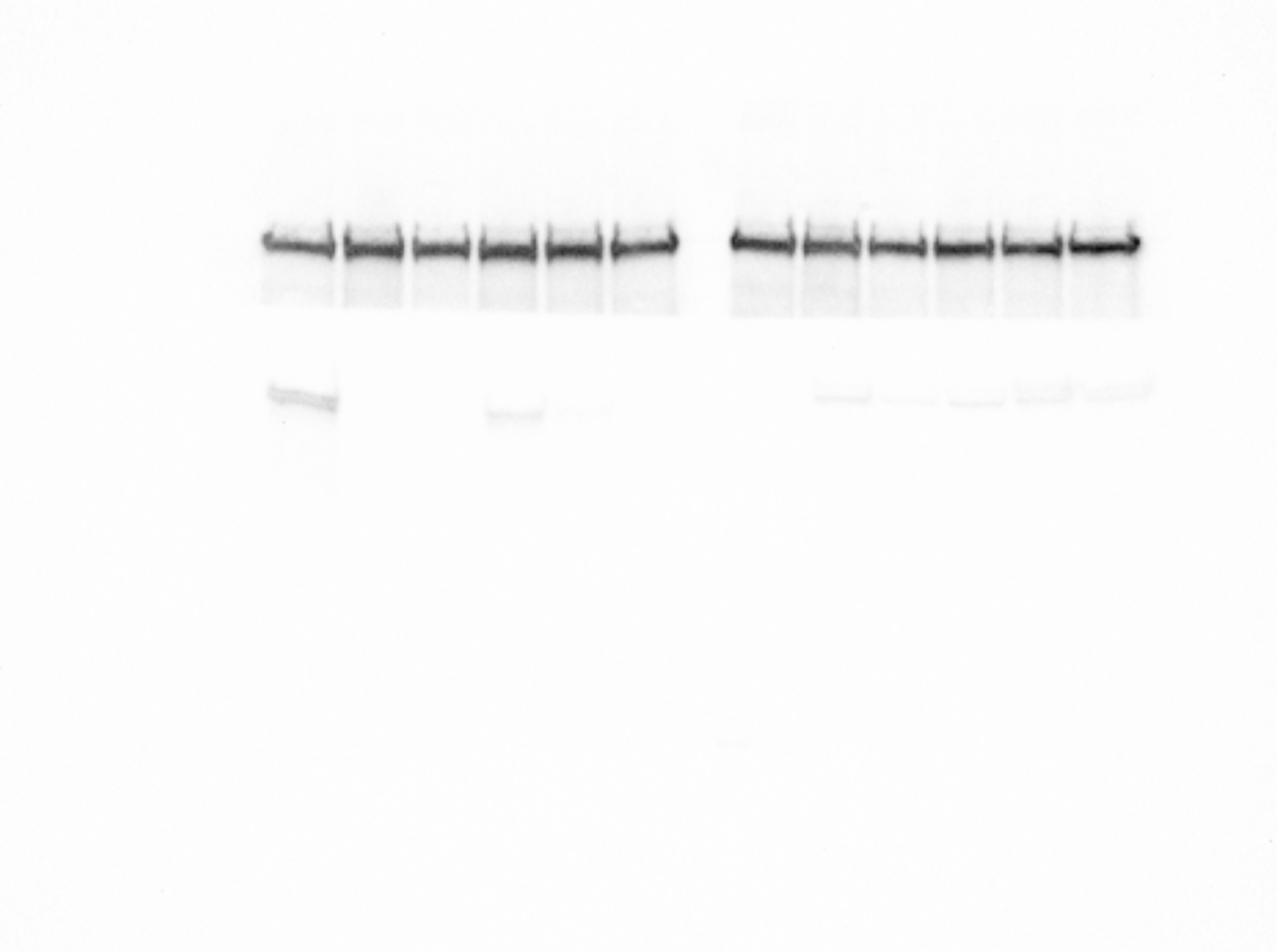

Supplement: Figure 2—figure supplement 1—source data 3. [file elife-72316-fig2-figsupp1-data3.zip › Figure2-figure supplement 1-source data 3.tif]

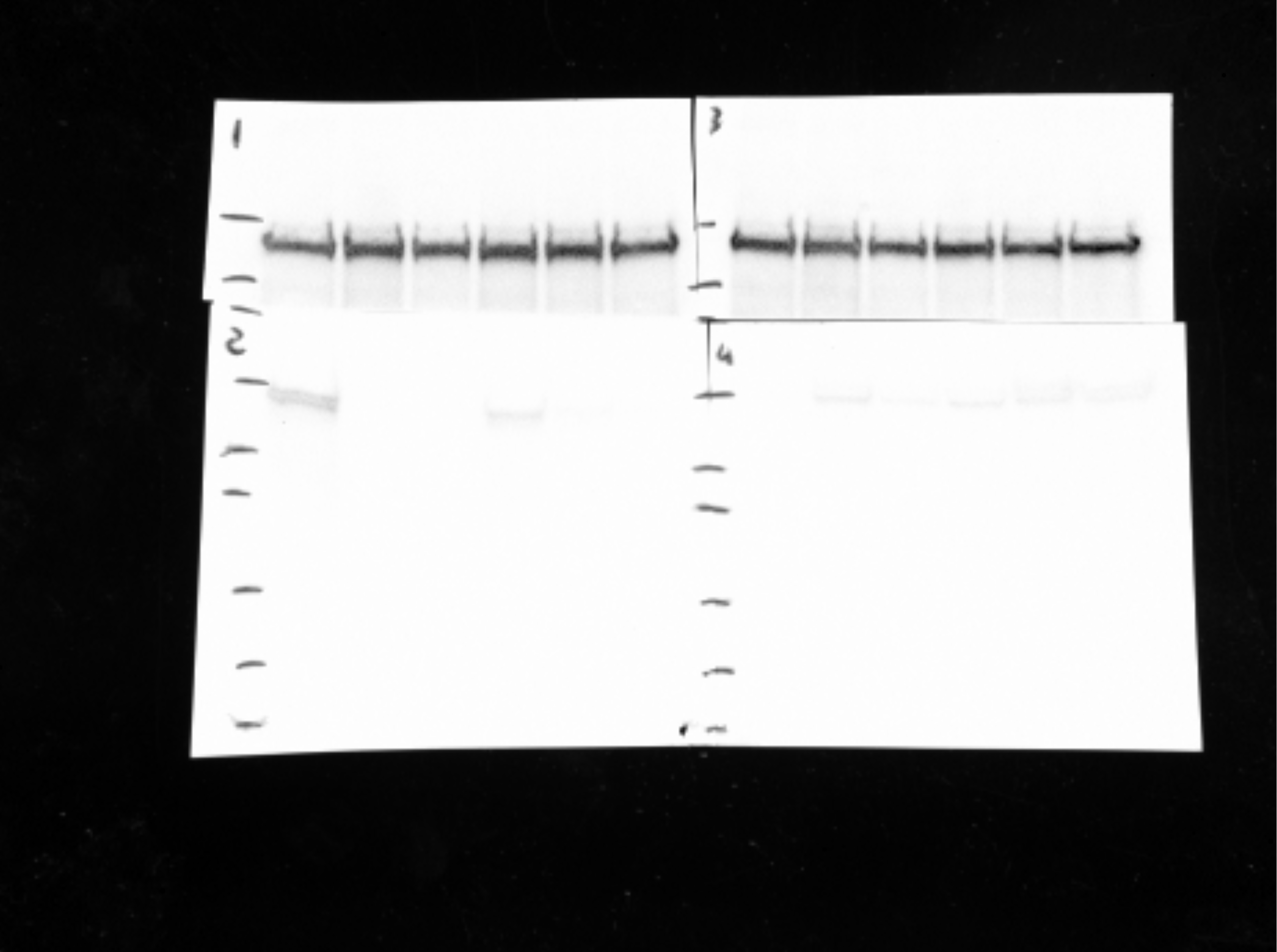

Supplement: Figure 2—figure supplement 1—source data 4. [file elife-72316-fig2-figsupp1-data4.zip › Figure2-figure supplement 1-source data 4.tif]

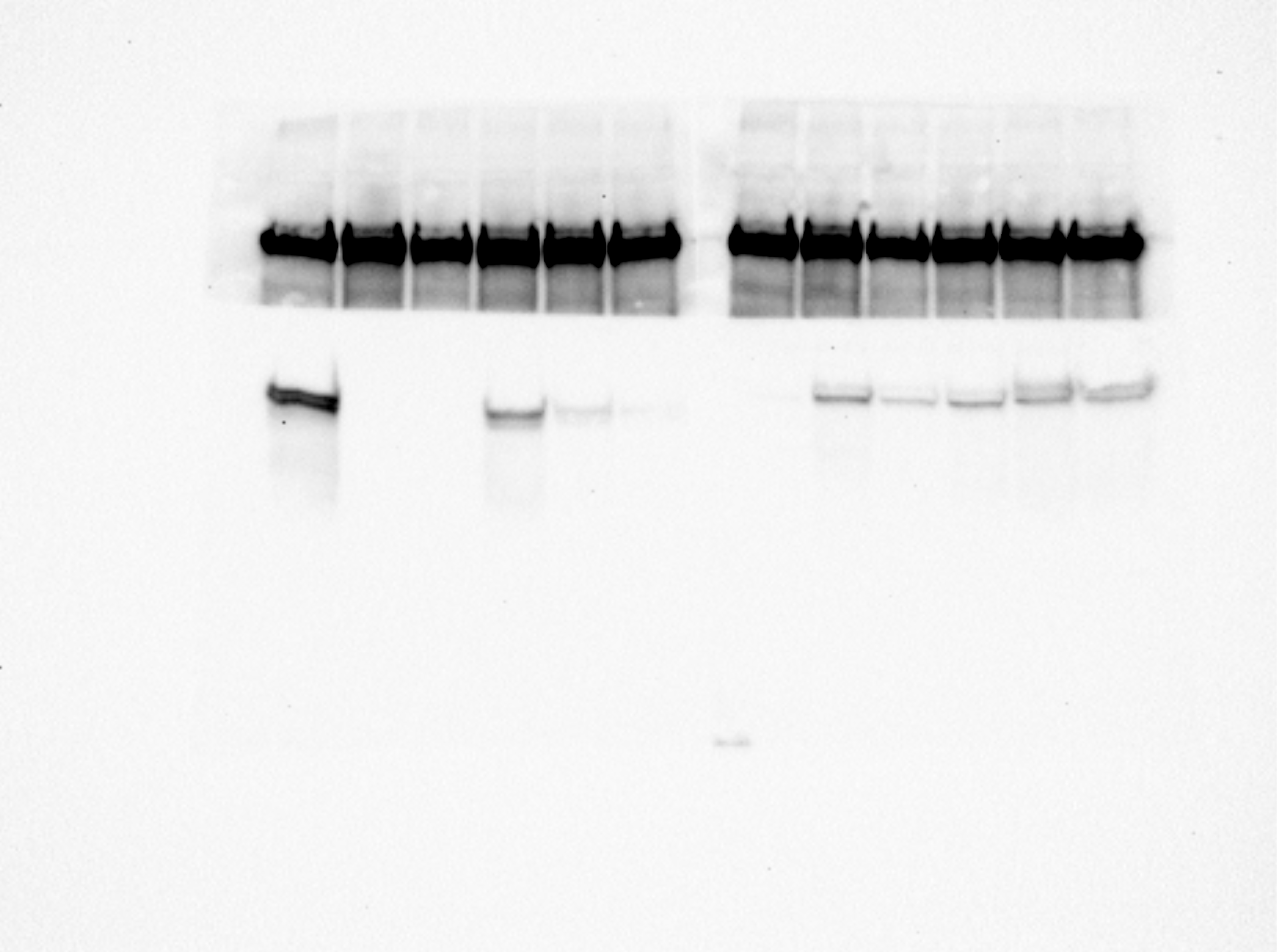

Supplement: Figure 2—figure supplement 1—source data 5. [file elife-72316-fig2-figsupp1-data5.zip › Figure2-figure supplement 1-source data 5.tif]

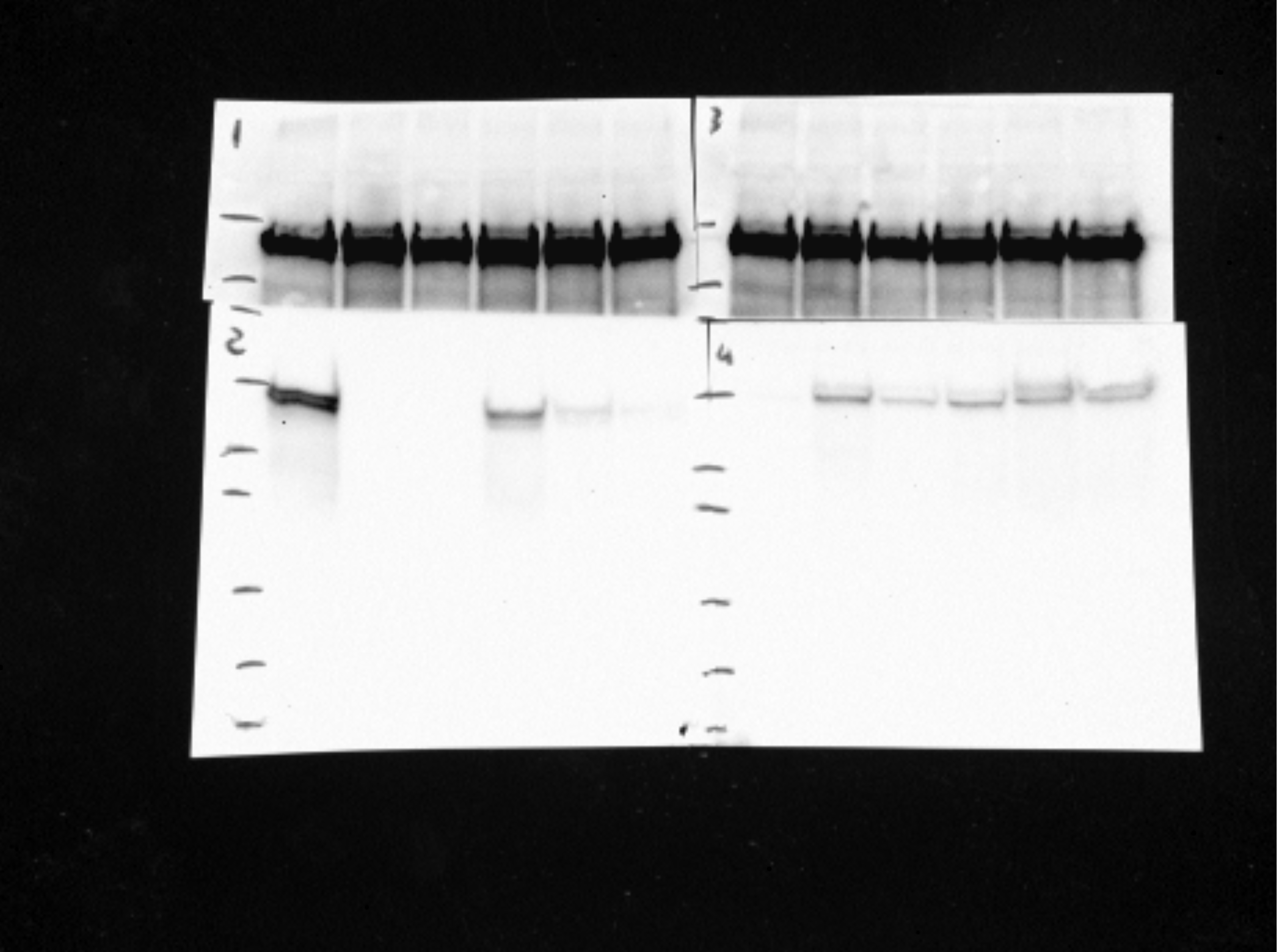

Supplement: Figure 2—figure supplement 1—source data 6. [file elife-72316-fig2-figsupp1-data6.zip › Figure2-figure supplement 1-source data 6.tif]

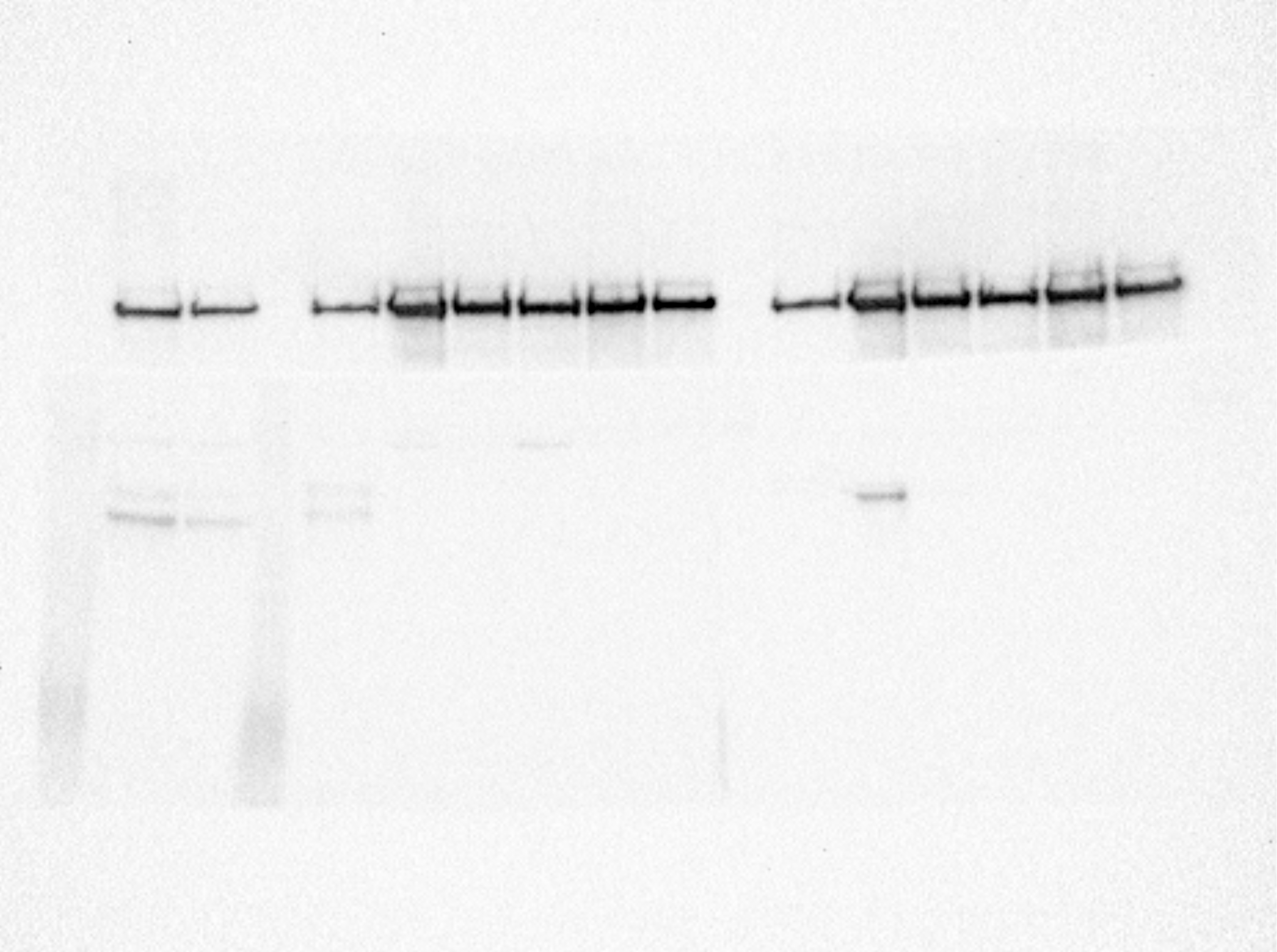

Supplement: Figure 2—figure supplement 1—source data 8. [file elife-72316-fig2-figsupp1-data8.zip › Figure2-figure supplement 1-source data 8.tif]

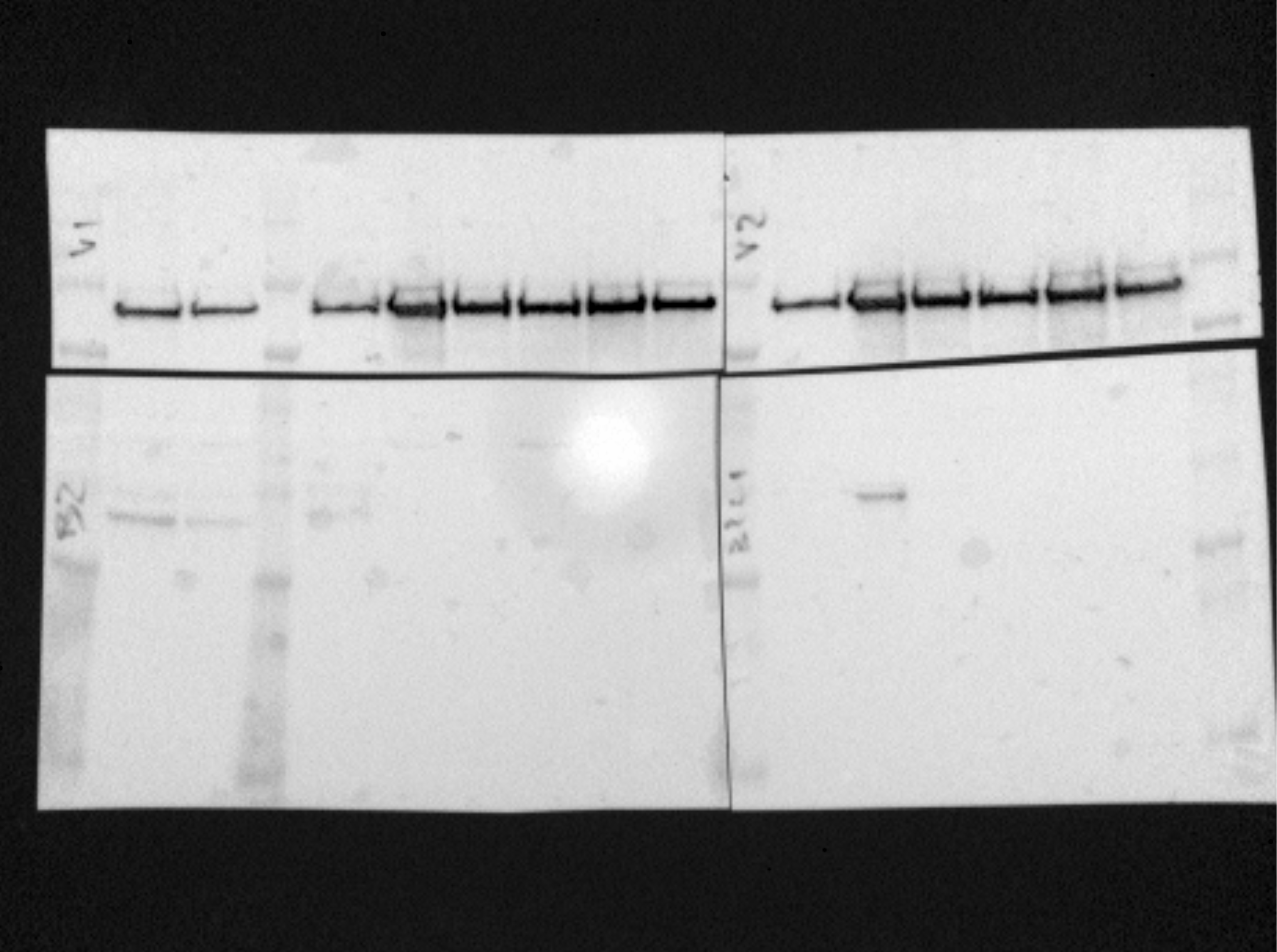

Supplement: Figure 2—figure supplement 1—source data 9. [file elife-72316-fig2-figsupp1-data9.zip › Figure2-figure supplement 1-source data 9.tif]

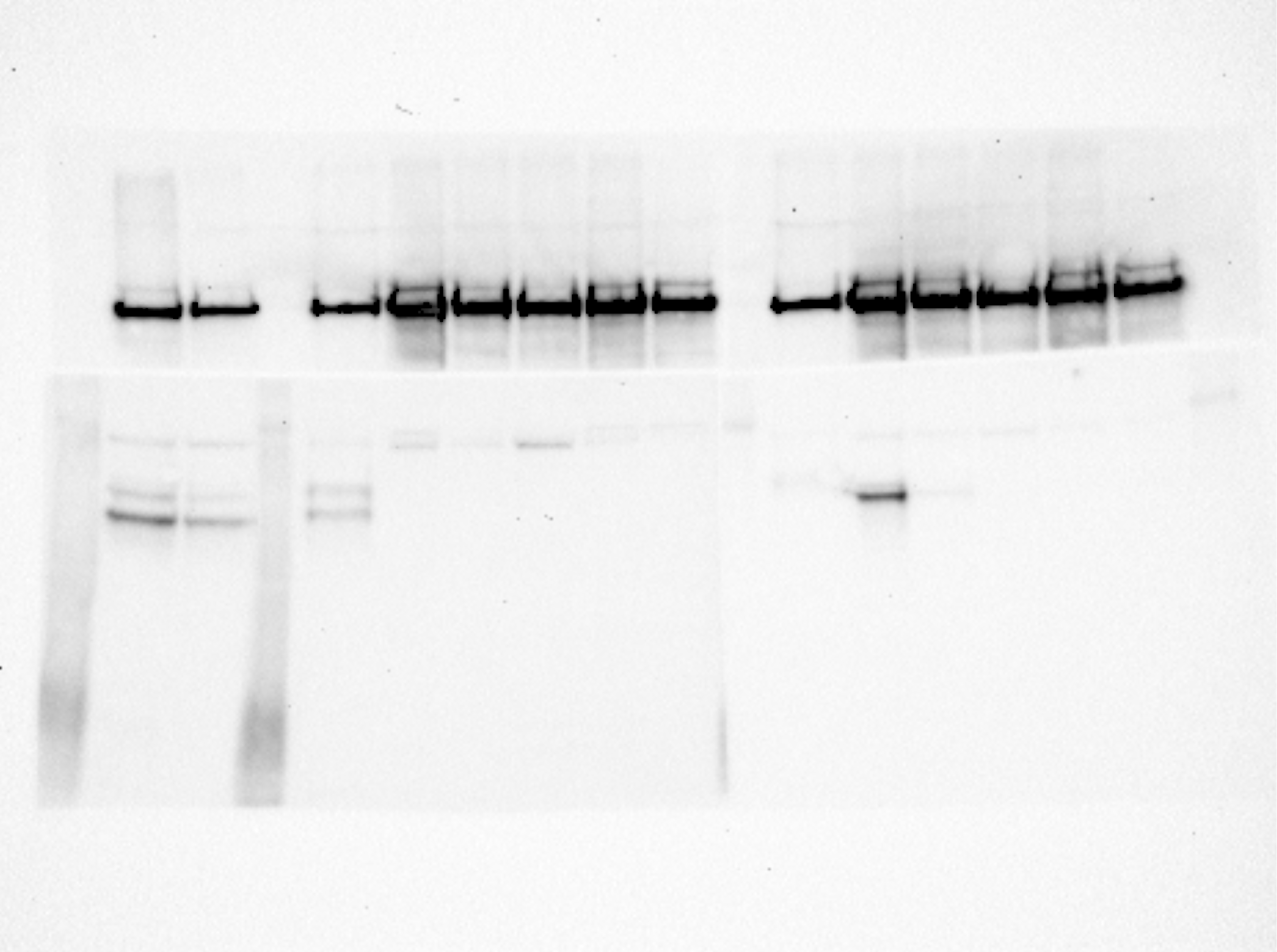

Supplement: Figure 2—figure supplement 1—source data 10. [file elife-72316-fig2-figsupp1-data10.zip › Figure2-figure supplement 1-source data 10.tif]

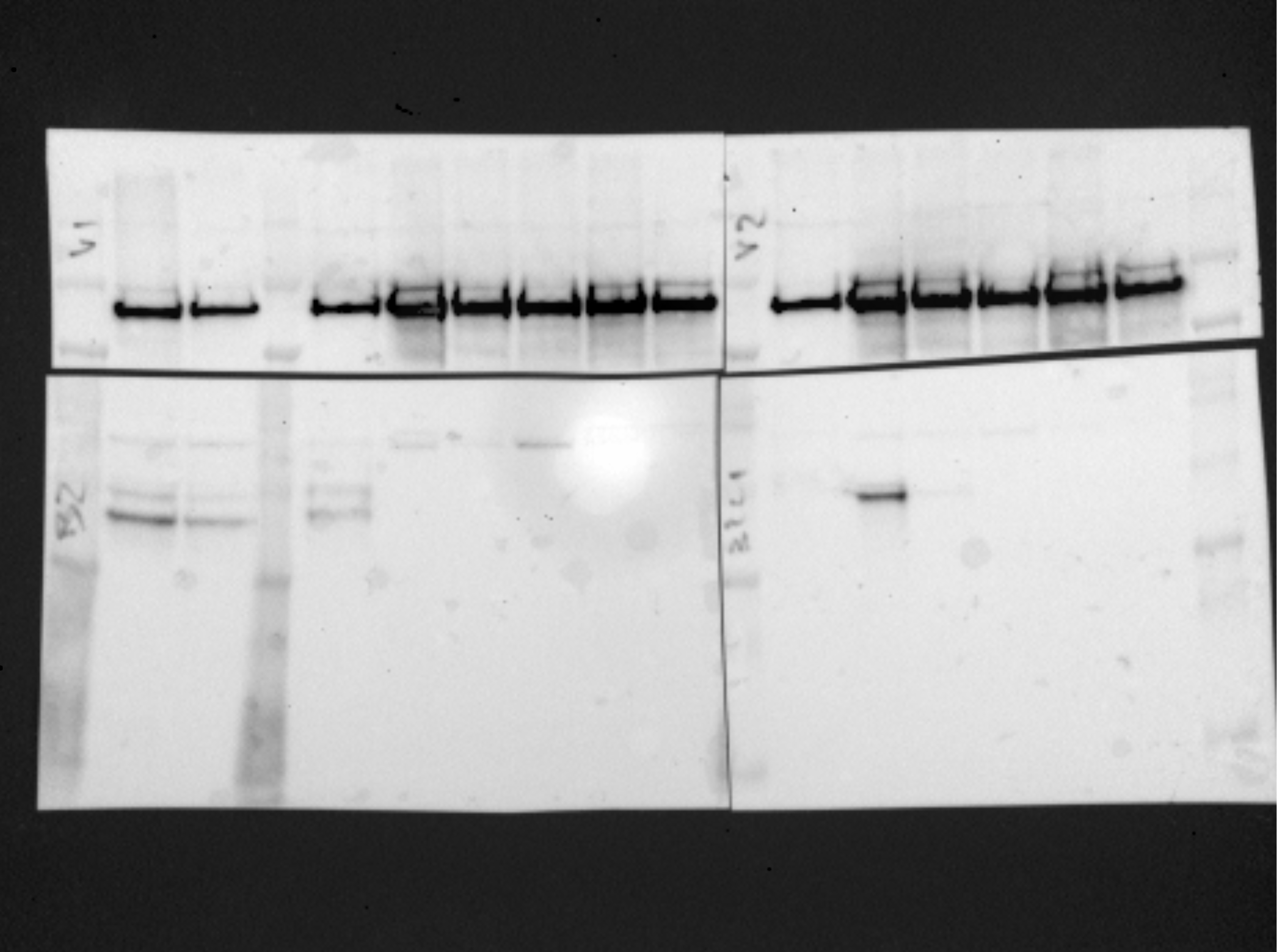

Supplement: Figure 2—figure supplement 1—source data 11. [file elife-72316-fig2-figsupp1-data11.zip › Figure2-figure supplement 1-source data 11.tif]

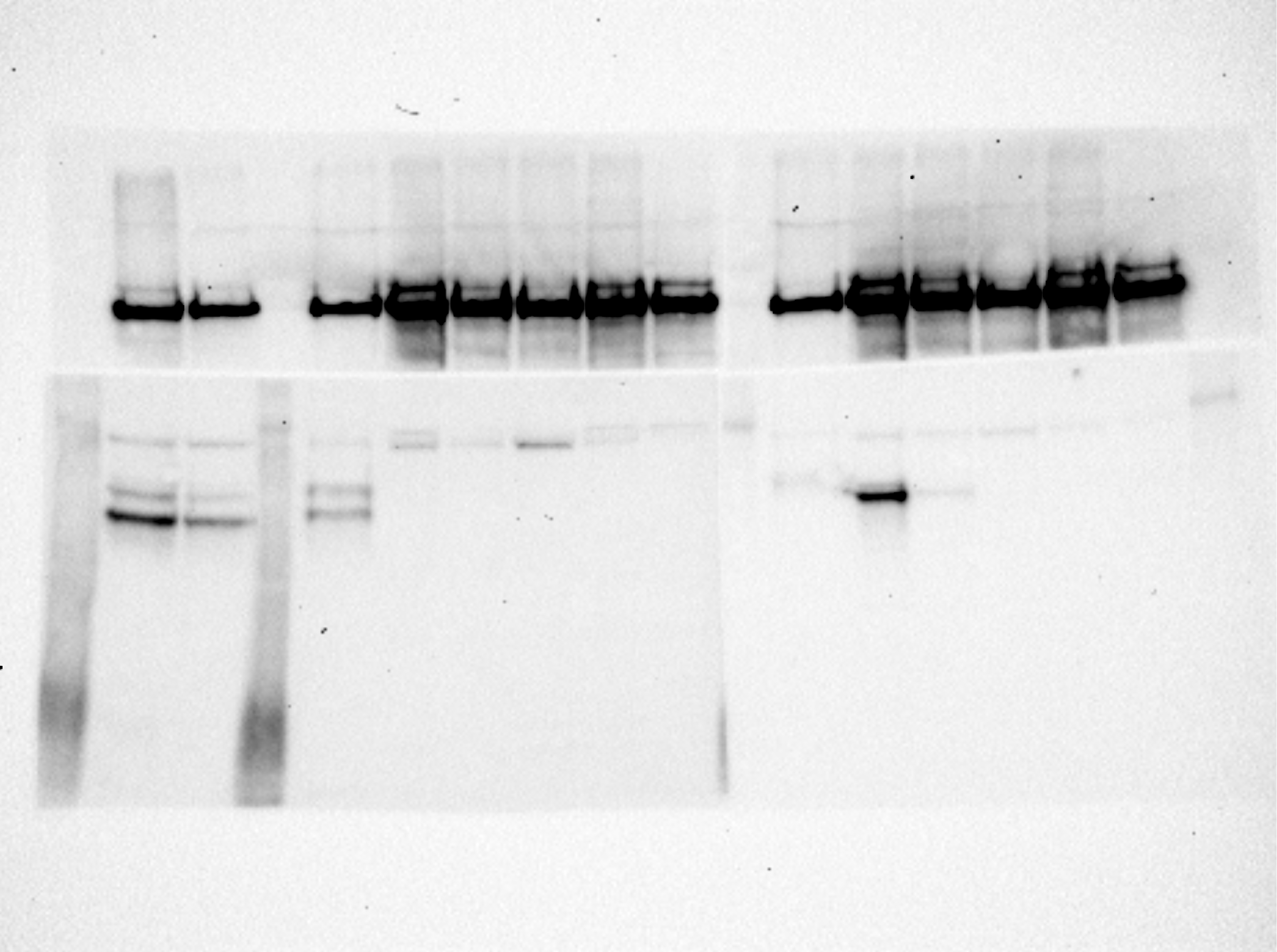

Supplement: Figure 2—figure supplement 1—source data 12. [file elife-72316-fig2-figsupp1-data12.zip › Figure2-figure supplement 1-source data 12.tif]

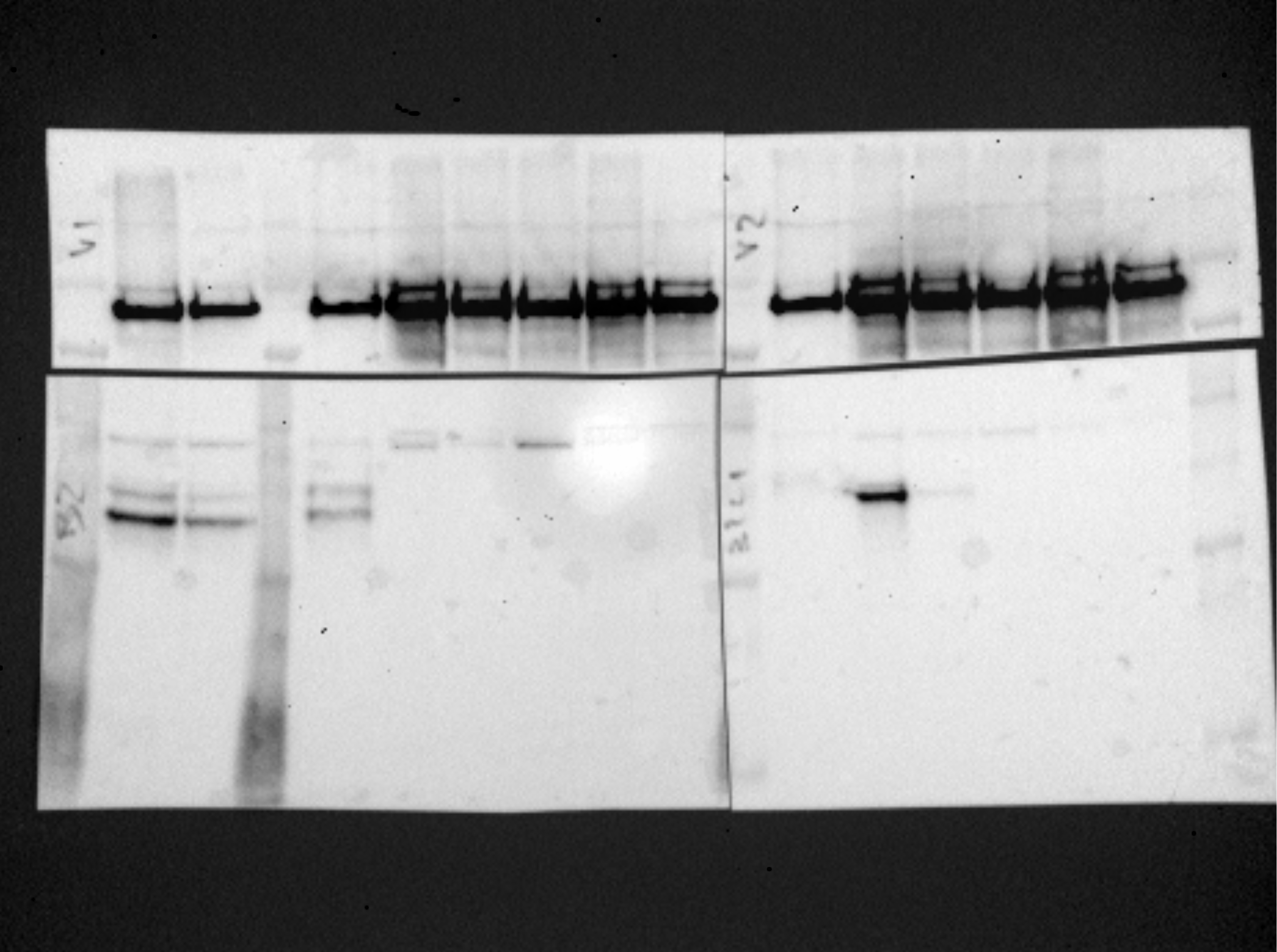

Supplement: Figure 2—figure supplement 1—source data 13. [file elife-72316-fig2-figsupp1-data13.zip › Figure2-figure supplement 1-source data 13.tif]

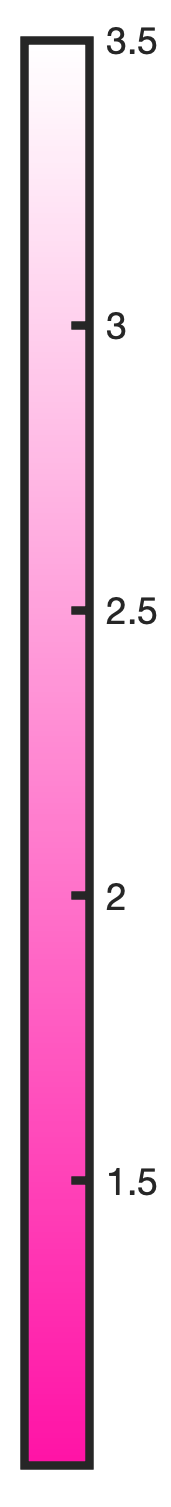

Supplement: Source code 1. [file elife-72316-code1.zip › Supplementary_Software_Elife/_cmap_mag.png]

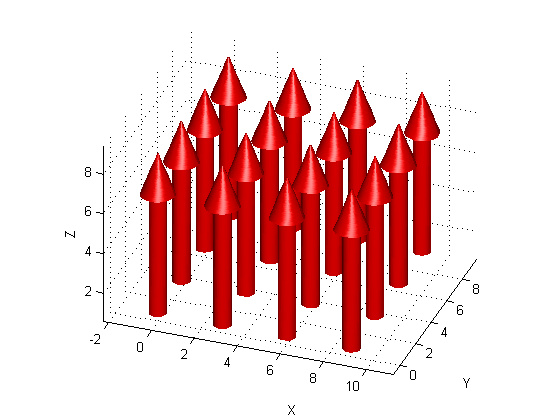

Supplement: Source code 1. [file elife-72316-code1.zip › Supplementary_Software_Elife/quiver3D_pub/html/demoQuiver3D_02.png]

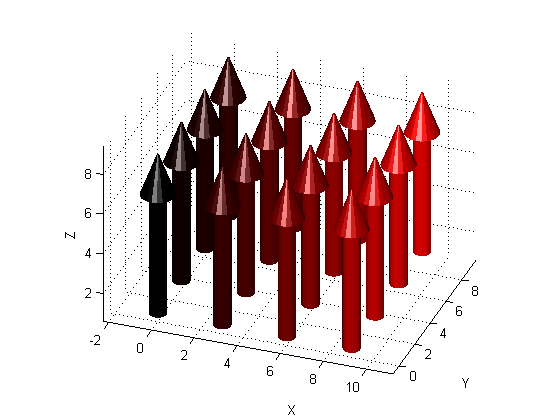

Supplement: Source code 1. [file elife-72316-code1.zip › Supplementary_Software_Elife/quiver3D_pub/html/demoQuiver3D_03.png]

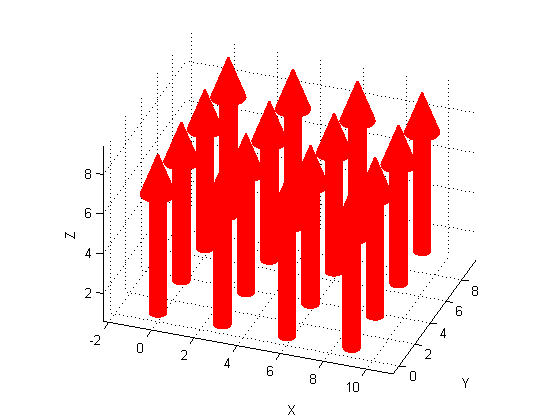

Supplement: Source code 1. [file elife-72316-code1.zip › Supplementary_Software_Elife/quiver3D_pub/html/demoQuiver3D_01.png]

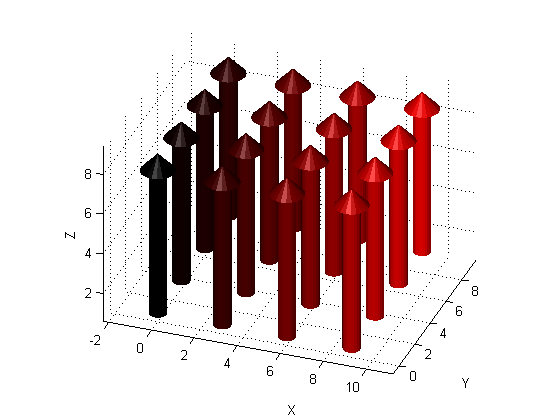

Supplement: Source code 1. [file elife-72316-code1.zip › Supplementary_Software_Elife/quiver3D_pub/html/demoQuiver3D_04.png]

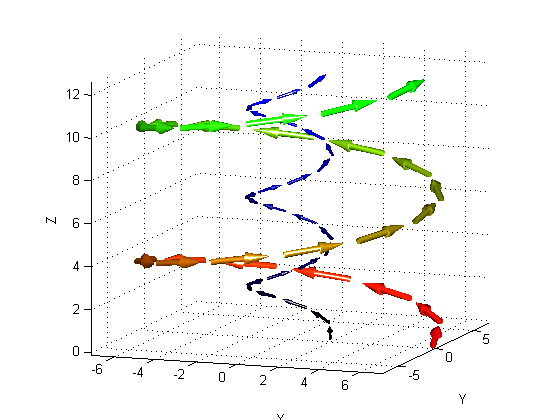

Supplement: Source code 1. [file elife-72316-code1.zip › Supplementary_Software_Elife/quiver3D_pub/html/demoQuiver3D_05.png]

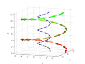

Supplement: Source code 1. [file elife-72316-code1.zip › Supplementary_Software_Elife/quiver3D_pub/html/demoQuiver3D.png]
