# Supplementary material for: A mechanosensing mechanism controls plasma membrane shape homeostasis at the nanoscale
Source: eLife. 2023 Sep 25;12:e72316. doi: 10.7554/eLife.72316 (PMC10569792; doi:10.7554/eLife.72316)

10 seconds exposure

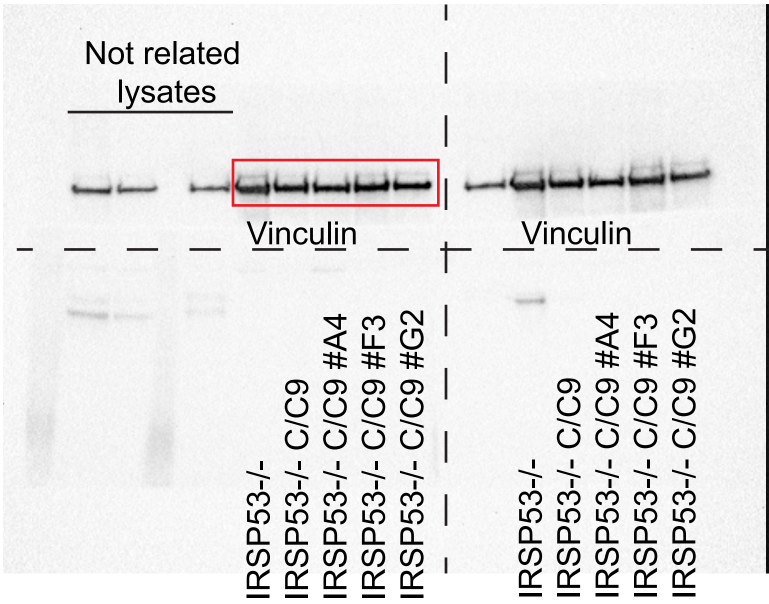

Marker overlay

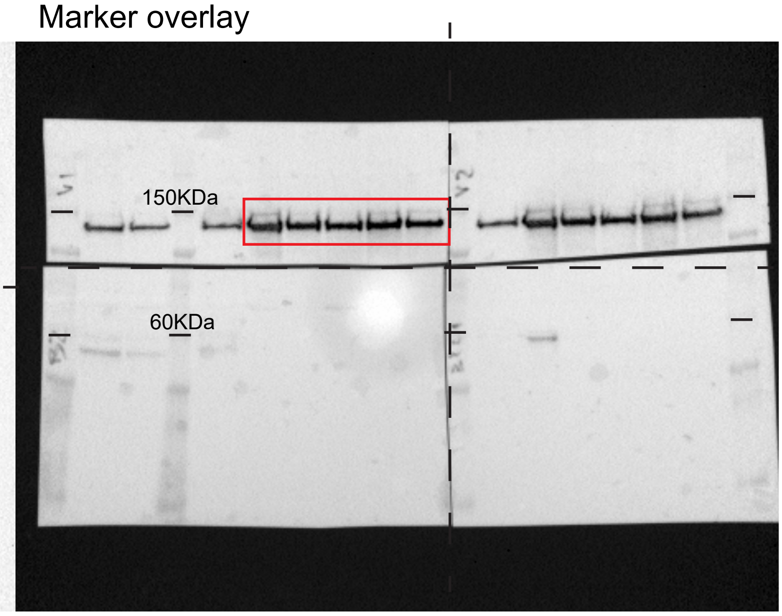

160 seconds exposure

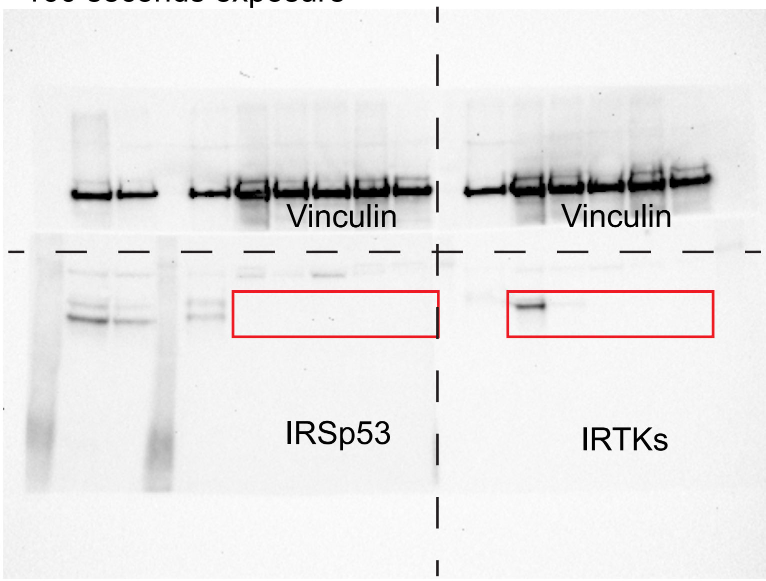

Marker overlay

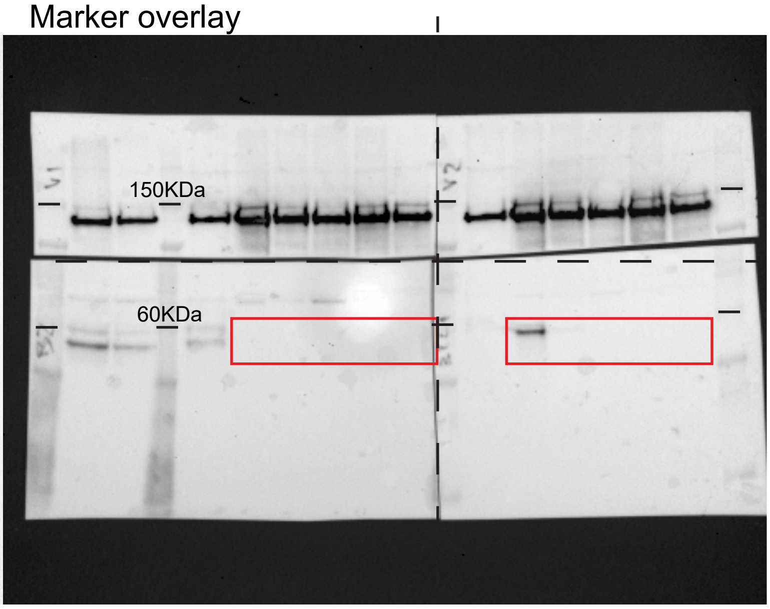

160 seconds exposure

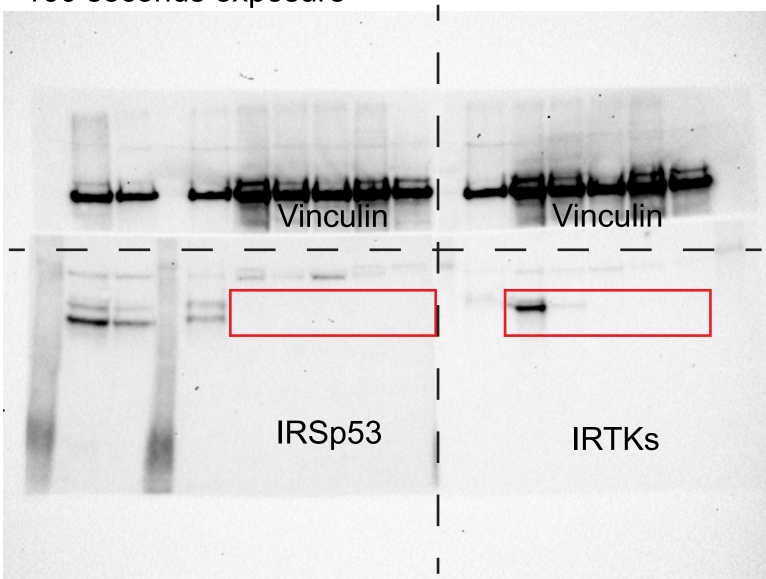

Marker overlay

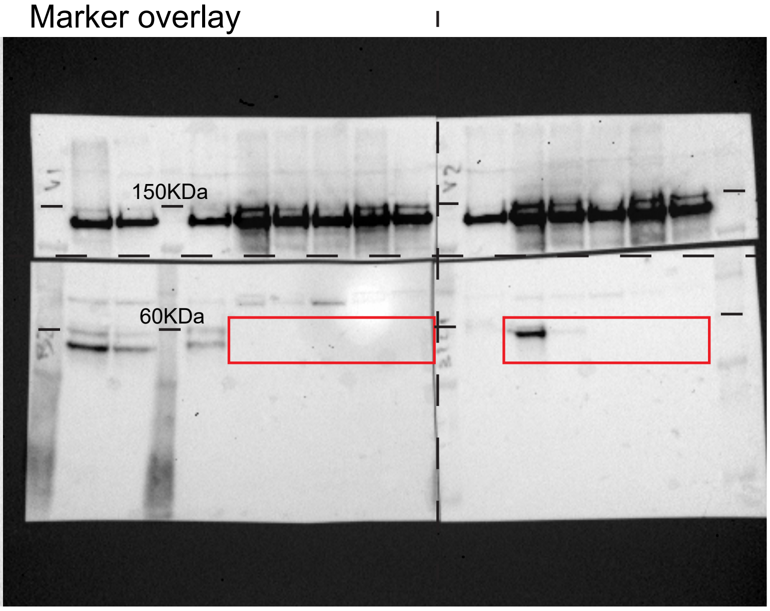

Supplement: Figure 2—figure supplement 1—source data 7. [file elife-72316-fig2-figsupp1-data7.pdf]
