# Supplementary material for: A mechanosensing mechanism controls plasma membrane shape homeostasis at the nanoscale
Source: eLife. 2023 Sep 25;12:e72316. doi: 10.7554/eLife.72316 (PMC10569792; doi:10.7554/eLife.72316)
Supplement: Source code 1. [file elife-72316-code1.zip › Supplementary_Software_Elife/README.pdf]

Supplementary Software Information

1 Code Organization

The code computes the chemo-mechanical response of lipid membranes with diffusing I-BAR proteins and interacting with actin cortex that can be contractile and apply drag forces on it and/or extensile and apply pushing forces on it.

The code is organized as follows:

- "Main Script.m" is the main script used to simulate the dynamics of the system
- "Post Process.m" is the script to post-process the output files
- "Input Files" folder: input files used in "Main Script.m" with different preexisting membrane templates
- "Functionals" folder: custom functions
- "Interpolation Geometry" folder: auxiliary calculations and structure for B-spline bases
- "Matrices" folder: Residual and Jacobian matrices for the Newton Raphson method used to solve the binding-diffusion of proteins
- "Reparametrization" folder: custom functions used to reparametrize the geometry
- "quiver3D pub" folder: Files for post processing and plotting velocity field of cortex
- "Output Files" folder: output of "Main Script.m"

2 Instructions for use

2.1 Main Script

The "Main Script.m" run with MATLAB2023a and it allows to compute the chemo-mechanical interaction between a lipid membrane, curving I-BAR proteins and actin cortex. The expected output is the dynamical reshaping of lipid membranes due to diffusion based sorting of I-BAR proteins on curved membrane templates which through regulatory specie

triggers gradient in contractility of the actin cortex and concomitant formation of extensile branched actin. The two architectures of actin compete to determine the fate of protruded membrane bud. The expected run time on a "normal" desktop computer can range from 10s of minutes to hours depending upon the choice of mesh and parameters.

The logical steps for the whole process are listed below:

1. Load input data (pre-generated membrane template, saved in the "Input Files" folder)
2. Definition of state variables, initial conditions, boundary conditions and parameters
3. Definition of solver options and B-spline order
4. Loop to solve the coupled PDEs in a staggered manner
   - Newton-Raphson method to solve protein concentration (ϕ)for a given membrane shape
   - Obtain the concentration field for regulatory specie (ψ) using direct solver approach
   - Obtain the velocity distribution of actin cortex which will apply drag force onto the membrane using direct solver approach
   - Matlab's in-built function "fmincon" to solve for membrane shape
   - Adaptive the time step depending upon rate of evolution of membrane shape
   - Reparametrization depending on the speed of the curve
   - Visualization of state variables after the iteration
5. Save the output file

2.2 Post Process

The "Post Process.m" runs with MATLAB2023a and it allows to post-process the output files. The logical steps for the whole process are listed below:

1. Load output data (saved in the "Output Files" folder)
2. Print the evolution of the system
3. Print membrane shape with colour coding of protein concentration and arrows for velocity vector of actin cortex.
