# Supplementary material for: A mechanosensing mechanism controls plasma membrane shape homeostasis at the nanoscale
Source: eLife. 2023 Sep 25;12:e72316. doi: 10.7554/eLife.72316 (PMC10569792; doi:10.7554/eLife.72316)
Supplement: Source code 1. [file elife-72316-code1.zip › Supplementary_Software_Elife/quiver3D_pub/html/demoQuiver3D.html]

quiver3D - Output a collection of arrows with various color and shape options


 


# quiver3D - Output a collection of arrows with various color and shape options

## Contents

- Syntax
- Description
- Example: Basic Call
- Lighting Effects
- Arrow-specific colors
- Change of stemRatios
- Helix Example
- Credits

## Syntax

- quiver3D(posArray, magnitudeArray)
- quiver3D(posArray, magnitudeArray, one\_ShortNameColor)
- quiver3D(posArray, magnitudeArray, one\_LongNameColor)
- quiver3D(posArray, magnitudeArray, one\_RGBvalueColor)
- quiver3D(posArray, magnitudeArray, many\_RGBvalueColor)

## Description

- quiver3D(posArray, magnitudeArray) - plot an arrow for each row of posArray in form of (x,y,z) with delta values corresponding
  to the rows of magnitudeArray (u,v,w) using arrow3D, which allows for a
  three-dimensional arrow representation. Since arrow3D uses 'surf', you may use 'camlight'
  and 'lighting' to add more powerful visual effects of the data.

- quiver3D(..., one\_ShortNameColor) - colors all arrows the same color using MATLAB's ShortName color convention, {'r','g',...}
  as per ColorSpec.

- quiver3D(..., one\_LongNameColor) - colors all arrows the same color using MATLAB's LongName color convention, {'red','green',...}
  as per ColorSpec.

- quiver3D(..., one\_RGBvalueColor) - colors all arrows the same color using the three element vector representation. For example
  [0, 1, 0.5]

- quiver3D(..., many\_RGBvalueColor) - a distinct color is assigned each of the individual arrows in the quiver in Nx3 format.

## Example: Basic Call

```
    [X, Y] = meshgrid(0:3:9, 0:3:9);
    Z = ones(size(X));
    U = zeros(size(X));
    V = U;
    W = ones(size(X))*8;
    posArray = [X(:),Y(:),Z(:)];
    magnitudeArray = [U(:),V(:),W(:)];
    quiverHandle = quiver3D(posArray, magnitudeArray, 'r');

    hold on;
    axis equal;
    grid on;
    xlabel('X'); ylabel('Y'); zlabel('Z');
    view(20,30);
```

## Lighting Effects

```
    lighting phong;
    camlight head;
```

## Arrow-specific colors

```
    numArrows = size(posArray,1);
    arrowColors = zeros(numArrows, 3);
    Rstream = (0:1/(numArrows-1):1)';
    arrowColors(:,1) = Rstream;
    delete(quiverHandle);
    colorQuiverHandle = quiver3D(posArray, magnitudeArray, arrowColors);
```

## Change of stemRatios

```
    delete(colorQuiverHandle);
    colorQuiverHandle = quiver3D(posArray, magnitudeArray, arrowColors, 0.9);
```

## Helix Example

```
    radius = 7;   height = 1;  numRotations = 2;  numPoints = 25;  arrowScale = 0.8;
    [posArray1, magnitudeArray1] = helix(radius, height, numRotations, numPoints, arrowScale);
    arrowColors1 = zeros(numPoints, 3);
    BlackToWhite = (0:1/(numPoints-1):1);
    WhiteToBlack = (1:-1/(numPoints-1):0);
    arrowColors1(:,1) = WhiteToBlack';
    arrowColors1(:,2) = BlackToWhite';

    radius = 2;   height = 0.66;  numRotations = 3;
    [posArray2, magnitudeArray2] = helix(radius, height, numRotations, numPoints, arrowScale);
    arrowColors2 = zeros(numPoints, 3);
    arrowColors2(:,3) = BlackToWhite';

    delete(colorQuiverHandle);
    quiver3D(posArray1, magnitudeArray1, arrowColors1, 0.6);
    quiver3D(posArray2, magnitudeArray2, arrowColors2, 0.6);
    axis equal;
    grid on;
    xlabel('X'); ylabel('Y'); zlabel('Z');
    view(20,10);
    axis tight;
    camlight head;
    lighting phong;
```

## Credits

Author: Shawn Arseneau

Created: September 15, 2006

Published with MATLAB® 7.0.1
